# The economic burden of excessive sugar consumption in Canada: should the scope of preventive action be broadened?

**DOI:** 10.17269/s41997-022-00615-x

**Published:** 2022-03-16

**Authors:** Siyuan Liu, Lalani L. Munasinghe, Katerina Maximova, Jennifer P. Taylor, Arto Ohinmaa, Paul J. Veugelers

**Affiliations:** 1 Population Health Intervention Research Unit, School of Public Health, University of Alberta, Edmonton, Alberta Canada; 2 MAP Centre for Urban Health Solutions, St. Michael’s Hospital, Toronto, Ontario Canada; 3 Dalla Lana School of Public Health, University of Toronto, Toronto, Ontario Canada; 4 Department of Applied Human Sciences, University of Prince Edward Island, Charlottetown, Prince Edward Island Canada; 5 School of Public Health, University of Alberta, 3-50 University Terrace, Edmonton, AB T6G 2T4 Canada

**Keywords:** Sugar, Nutrition, Public health, Disease prevention, Chronic diseases, Economic burden, Taxation—health care costs, Health policy, Sucre, nutrition, santé publique, prévention des maladies, maladies chroniques, fardeau économique, taxation—coûts des soins de santé, politique de santé

## Abstract

**Objective:**

Excessive sugar consumption is an established risk factor for various chronic diseases (CDs). No earlier study has quantified its economic burden in terms of health care costs for treatment and management of CDs, and costs associated with lost productivity and premature mortality. This information, however, is essential to public health decision-makers when planning and prioritizing interventions. The present study aimed to estimate the economic burden of excessive free sugar consumption in Canada.

**Methods:**

Free sugars refer to all monosaccharides and disaccharides added to foods plus sugars naturally present in honey, syrups, and fruit juice. Based on free sugar consumption reported in the 2015 Canadian Community Health Survey–Nutrition and established risk estimates for 16 main CDs, we calculated the avoidable direct health care costs and indirect costs.

**Results:**

If Canadians were to comply with the free sugar recommendation (consumption below 10% of total energy intake (TEI)), an estimated $2.5 billion (95% CI: 1.5, 3.6) in direct health care and indirect costs could have been avoided in 2019. For the stricter recommendation (consumption below 5% of TEI), this was $5.0 billion (95% CI: 3.1, 6.9).

**Conclusion:**

Excessive free sugar in our diet has an enormous economic burden that is larger than that of any food group and 3 to 6 times that of sugar-sweetened beverages (SSBs). Public health interventions to reduce sugar consumption should therefore consider going beyond taxation of SSBs to target a broader set of products, in order to more effectively reduce the public health and economic burden of CDs.

## Résumé

**Objectif:**

La consommation excessive de sucre est un facteur de risque connu pour diverses maladies chroniques. Aucune étude antérieure n’en a chiffré le fardeau économique en termes de coûts de soins de santé pour le traitement et la prise en charge des maladies chroniques, et de coûts associés à la perte de productivité et à la mortalité prématurée. Ces informations sont pourtant essentielles aux décideurs de la santé publique pour planifier les interventions et gérer les priorités. Notre étude a cherché à estimer le fardeau économique de la consommation excessive de sucres libres au Canada.

**Méthode:**

Les sucres libres désignent tous les monosaccharides et disaccharides ajoutés aux aliments, plus les sucres naturellement présents dans le miel, les sirops et les jus de fruits. D’après la consommation de sucres libres constatée dans l’Enquête sur la santé dans les collectivités canadiennes – Nutrition de 2015 et les estimations du risque connues pour 16 grandes maladies chroniques, nous en avons calculé les coûts évitables directs (de soins de santé) et indirects.

**Résultats:**

Si les Canadiens s’en tenaient à la consommation recommandée de sucres libres (soit moins de 10 % de l’apport énergétique total [AÉT]), il est estimé que 2,5 milliards de dollars (IC de 95 % : 1,5, 3,6) en coûts directs de soins de santé et en coûts indirects auraient pu être évités en 2019. Si la recommandation plus stricte (consommation inférieure à 5 % de l’AÉT) avait été respectée, les coûts évités se seraient chiffrés à 5 milliards de dollars (IC de 95 % : 3,1, 6,9).

**Conclusion:**

L’excès de sucres libres dans notre régime représente un fardeau économique énorme, supérieur à celui de tout autre groupe d’aliments et de 3 à 6 fois supérieur à celui des boissons édulcorées au sucre (BÉS). Il faudrait donc envisager des interventions en santé publique visant à réduire la consommation de sucre qui vont au-delà de la taxation des BÉS pour cibler un panier élargi de produits afin de réduire plus efficacement le fardeau sanitaire et économique des maladies chroniques.

## Introduction

Chronic diseases (CDs) are a leading cause of death in the world (WHO, 2015a). In Canada, CDs, including diabetes, cardiovascular diseases (CVD), and cancer, accounted for 62% of all deaths in 2019 (Statistic Canada, 2021). The treatment and management of CDs were estimated to consume 67% of all direct health care costs, adding up to CA $190 billion annually (Elmslie, 2012).

Adopting healthy lifestyles, such as healthy eating, active living, tobacco abstinence, and responsible alcohol consumption, can prevent up to 80% of type 2 diabetes and CVD and 40% of cancers (Lieffers et al., 2018). Among the aforementioned four major lifestyle risk factors for CDs, an unhealthy diet has been shown to have the largest burden (WHO, 2015a). Despite a series of healthy eating recommendations issued in Canada to improve health and reduce CDs (Health Canada, 2019; Health Canada, 2007), the majority of Canadian residents do not meet these recommendations (Liu et al., 2020; Black & Billette, 2013). For example, more than three out of four Canadians do not consume enough vegetables and fruit (Black & Billette, 2013; Ekwaru et al., 2016), and nearly two out of three consume more free sugar than what is recommended (Liu et al., 2020).

The World Health Organization (WHO) defines free sugars as: “all monosaccharides and disaccharides added to foods by the manufacturer, cook or consumer, plus sugars naturally present in honey, syrups and fruit juice” (WHO, 2015b). They recommend the consumption of free sugars to be below 10% of the daily total energy intake (TEI), and ideally below 5% of TEI (WHO, 2015b). Recently, we reported that only 33.8% and 5.4% of Canadian residents met these free sugar recommendations of below 10% and 5% of TEI, respectively, in 2015 (Liu et al., 2020).

Considering the financial pressures on health care systems, understanding the magnitude of avoidable costs for the treatment and management of CDs is essential to public health decision-makers. Canadian research to date has revealed the economic burden of inadequate intake of vegetables and fruit (Ekwaru et al., 2016), dairy products (McCarron et al. 2004), dietary fibre (Abdullah et al., 2015), and other healthful foods and food groups (Lieffers et al., 2018; Loewen et al., 2019). Canadian research has also revealed the economic burden associated with excess consumption of harmful foods and specifically sugar-sweetened beverages (SSBs) and sugary drinks (Jones et al., 2017; Lieffers et al., 2018; Loewen et al., 2019). Considering that only 17.5% of free sugar in the Canadian diet originates from SSBs (Liu et al., 2020), the economic burden attributable to free sugar from *all* foods and beverages in our diet is likely much higher than that of SSBs, and therefore more relevant to public health decision-makers. However, no study to date has estimated the economic burden of excessive free sugar consumption from all foods and beverages. This study quantifies the direct health care costs (hospital, physician, and drug) and indirect costs that could be avoided if Canadians comply with existing free sugar consumption recommendations.

## Methods

We established a methodological approach to quantify the economic burden of the inadequate and excessive consumption of foods and beverages (Ekwaru et al., 2016; Lieffers et al., 2018; Loewen et al., 2019). For the present study, we applied this approach to estimate the economic burden of excessive free sugar consumption. In brief, in this approach, we make use of the vigorous estimates from the Global Burden of Disease (GBD) report (GBD, 2015) of the risk for CDs associated with the consumption of free sugar and the free sugar consumption of Canadian residents (for ease of reporting, hereafter referred to as Canadians) to calculate population attributable fractions (PAF). A PAF represents the fraction of disease (CDs) that is avoided if a population avoids exposure to a certain risk factor: in this study, the excessive consumption of free sugar. Once we have estimated the fraction of CDs that can be avoided, we can calculate what costs can be avoided for the treatment and management of these CDs and lost productivity and premature mortality. Below, we provide a description of the approach. For full details, we refer to our earlier work (Ekwaru et al., 2016; Lieffers et al., 2018; Loewen et al., 2019).

### Risk for chronic diseases associated with free sugar consumption

We extracted age- and sex-specific relative risk estimates for CDs associated with the consumption of SSBs from the 2013 GBD report (GBD, 2015) in the absence of established risk estimates for CDs associated with free sugar consumption. We assumed that the risk associated with the consumption of free sugar in our diet is the same as the risk associated with the consumption of the equivalent amount of free sugar from SSBs. As the WHO recommendations state that the consumption of free sugars are not to exceed 5% or 10% of TEI (WHO, 2015b), we applied increments of 5% of TEI from free sugar as units for comparisons. We therefore adjusted the age- and sex-specific relative risks obtained from the GBD such that they applied to increments of 5% of TEI from free sugar. We included risk estimates for those 16 CDs for which cost information is available through the Economic Burden of Illness in Canada (EBIC) (Public Health Agency of Canada, 2018). These CDs include esophageal cancer, liver cancers, breast cancer, uterine cancer, colorectal cancer, pancreatic cancer, ovarian cancer, kidney cancer, thyroid cancer, leukemia, ischemic heart disease, ischemic stroke, hemorrhage stroke, diabetes, chronic kidney disease (CKD), and low back pain. The risk estimates for these CDs are listed elsewhere (Lieffers et al., 2018; Loewen et al., 2019).

### Free sugar consumption of Canadians

We accessed the 2015 Canadian Community Health Survey (CCHS) – Nutrition (Statistics Canada, 2018) and used recently published free sugar content estimates (Liu et al., 2020) to estimate the free sugar consumption of Canadians. The 2015 CCHS – Nutrition (response rate 61.6%) collected 24-h dietary recalls of 20,487 participants aged above 1 year living in the ten provinces in Canada, of whom 7608 completed a second 24-h dietary recall. The 24-h dietary recalls were administered using an Automated Multiple-Pass Method. Using both the first and second 24-h recall data, we estimated usual free sugar consumption and usual total energy intake (TEI) for each age and sex subgroup by applying the National Cancer Institute (NCI) method (NCI, 2020). Sampling weights that considered initial weights, non-response, and post-stratification (Statistics Canada, 2018) were applied to ensure estimates are representative of all Canadians. Using the SAS Macros of the NCI method, we obtained the proportion of each age and sex subgroup consuming 0–5%, 5–10%, 10–15%, 15–20%, 20–25%, and 25%+ TEI from free sugars per day. All statistical analyses were performed using SAS (version 9.4, SAS Institute) software.

### Avoidable chronic diseases

We calculated the fraction of diseases that could theoretically be avoided by reducing free sugar consumption to an amount below the free sugar recommendations (PAFs) for each of the 16 CDs and every age and sex sub-group based on the above-mentioned risk estimates and consumption levels. We used the method recommended by Krueger et al. (2013) that considers multiple risk exposure levels. The PAFs calculation equation is as follows:

$$ PAF=\frac{\sum_{i=1}^n{P}_i\left({RR}_i-1\right)}{1+{\sum}_{i=1}^n{P}_i\left({RR}_i-1\right)} $$

Where *P*_*i*_ is the proportion of people in interval *i*, *i* (interval) refers to the consumption of 0–5%, 5–10%, 10–15%, 15–20%, 20–25%, and 25%+ of TEI from free sugars, *RR* is the relative risk for each 5% increase in percentage of TEI from free sugar, ${RR}_i={RR}^{\left({X}_i-L\right)}$ is the relative risk for interval *i* relative to the recommendation considered (either 10% or 5% of TEI), *X*_*i*_ is the mid value of interval *i*, *L* is the recommendation considered, and *n* is the number of intervals above the recommendation considered.

### Avoidable health care costs and indirect costs

We considered hospital, physician, and drug costs for the treatment and management of CDs reported in the 2019 National Health Expenditure Trends (CIHI, 2019) and the age- and sex-specific proportions of each of the 16 CDs from the 2010 Economic Burden of Illness in Canada (PHAC 2018) to calculate the direct costs for each of the 16 CDs. We estimated the indirect costs of excessive free sugar intake using the human capital approach (Krueger et al., 2013). Following this approach, we extracted the ratios of indirect costs (costs associated with short- and long-term disability and with mortality) versus direct health care costs for each of the 16 CDs from the 1998 EBIC (Health Canada, 2002). We then multiplied these ratios by the 2019 direct health care costs for each age and sex group while assuming these ratios did not change over time and applying the disaggregation step from Krueger et al. (2013) to estimate the total avoidable costs. All costs were reported in 2019 Canadian dollars. We conducted a sensitivity analysis by recalculating the above while using the 95% confidence interval lower and upper boundary estimates for risk estimates extracted for the 2013 GBD report (GBD, 2015).

## Results

In Canada, women, on average, consumed 59.9 g of free sugar and 1510 kilocalories per day, while men consumed 75.3 g of free sugar and 2004 kilocalories per day (Table 1). The distribution of free sugar as a percentage of TEI was similar for both sexes with slightly more men (34.2%) than women (32.1%) adhering to the recommendation that free sugar consumption should not exceed 10% of TEI, and slightly more men (6.0%) than women (4.6%) adhering to the stricter recommendation that the consumption of free sugar should not exceed 5% of TEI (Table 1).

**Table 1:** The distribution of usual intake of free sugar, total energy, and the percentage of total energy from free sugar by sex in Canada, 2015

	Mean	Percentile							Recommendations	
		5th	10th	25th	50th	75th	90th	95th	<5% TEI	<10% TEI
Free sugar intake (grams/day)										
Women	59.9	17.3	22.6	34.4	52.5	77.2	106.5	127.5	-	-
Men	75.3	18.9	25.5	40.6	64.5	98.1	138.5	168.3	-	-
Women and men	67.1	17.7	23.7	37.0	58.0	87.2	122.1	147.8	-	-
TEI (kilocalories/day)										
Women	1510	746	880	1129	1455	1829	2213	2463	-	-
Men	2004	909	1091	1438	1907	2462	3044	3439	-	-
Women and men	1753	791	944	1249	1658	2152	2681	3040	-	-
TEI from free sugar (%)									-	-
Women	13.4	5.1	6.4	9.0	12.5	16.8	21.5	24.7	4.6	32.1
Men	13.2	4.7	6.0	8.7	12.3	16.8	21.6	24.9	6.0	34.2
Women and men	13.3	4.9	6.2	8.7	12.3	16.8	21.6	24.9	5.4	33.8

*TEI*, total energy intake

Table 2 shows the population attributable fractions, i.e., the estimated percentage of CDs that is avoided if Canadians avoid consuming free sugar in excess of recommendations. The estimates for diabetes (27.0% and 44.8% for the recommendations of <10% of TEI and <5% of TEI respectively) stood out as the highest of all CDs, followed by cardiovascular and cerebrovascular disease, i.e., ischemic heart disease, ischemic stroke, and hemorrhagic stroke combined (5.2% and 10.2% for the recommendations of <10% of TEI and <5% of TEI, respectively). The estimated percentage of diabetes and cardiovascular disease that is avoided when adhering to free sugar recommendations is similar for women and men (Table 2).

**Table 2:** The fractions (%) of chronic diseases that are avoided if Canadians would not consume free sugar in excess of recommendations

	The avoidable fractions (%) of diseases					
	<10% TEI from free sugar			<5% TEI from free sugar		
	Women	Men	Women and Men	Women	Men	Women and Men
Cancer						
Esophagus	0.9	0.8	0.8	1.7	1.6	1.7
Liver	0.5	0.7	0.6	1.0	1.3	1.2
Colorectal	0.2	0.4	0.3	0.4	0.8	0.6
Pancreas	0.2	0.2	0.2	0.5	0.4	0.4
Kidney	0.8	0.6	0.7	1.6	1.1	1.4
Thyroid	0.4	0.5	0.5	0.8	1.0	0.9
Leukemia	0.4	0.2	0.3	0.8	0.4	0.6
Post-menopausal breast	0.3	-	-	0.6	-	-
Uterus	1.5	-	-	2.9	-	-
Ovary	0.1	-	-	0.2	-	-
Cardiovascular diseases						
Ischemic heart disease	1.5	1.4	1.5	3.0	2.7	2.8
Ischemic stroke	1.7	1.5	1.6	3.3	3.0	3.2
Hemorrhagic stroke	2.3	2.0	2.1	4.4	3.9	4.2
Diabetes	27.0	27.2	27.0	44.7	45.1	44.8
Chronic kidney disease	1.6	1.4	1.5	3.1	2.7	2.9
Low back pain	0.3	0.2	0.3	0.6	0.5	0.5

*TEI*, total energy intake

The economic burden resulting from free sugar consumption above 10% of TEI was estimated to be $2.5 billion (95% CI: $1.5 to $3.6 billion) per year (Table 3). This amount included about $1.1 billion per year in direct health care costs for the treatment and management of CDs and $1.4 billion per year in indirect costs, i.e., costs associated with lost productivity and premature mortality. For the stricter recommendation, free sugar consumption above 5% of TEI caused an estimated economic burden of $5.0 billion (95% CI: $3.1 to $6.9 billion) per year which included $2.2 billion per year in direct health care costs and $2.7 billion per year in indirect costs (Table 3). Around 93% of the costs were attributable to diabetes ($2.3 billion and $4.6 billion for consuming free sugar in excess of 10% and 5% of TEI, respectively). Direct health care costs and indirect costs were substantially higher among men than among women (Table 3).

**Table 3:** The economic burden of excessive sugar consumption of Canadians by chronic disease, sex and age group in 2019

	Estimated direct health care costs and indirect costs in 2019					
	Free sugar consumption <10% TEI			Free sugar consumption <5% TEI		
	Direct costs	Indirect costs	Total	Direct costs	Indirect costs	Total
Chronic disease						
Cancer						
Esophagus	465,990	2,225,109	2,691,099	996,400	4,757,827	5,754,227
Liver	205,906	983,203	1,189,109	429,501	2,050,875	2,480,376
Colorectal	2,298,749	10,976,565	13,275,315	4,887,054	23,335,762	28,222,816
Pancreas	160,348	765,664	926,012	338,208	1,614,950	1,953,158
Kidney	601,615	2,872,719	3,474,334	1,253,400	5,985,003	7,238,403
Thyroid	390,938	1,866,736	2,257,674	782,896	3,738,343	4,521,240
Leukemia	1,046,917	4,999,048	6,045,965	2,045,863	9,769,027	11,814,889
Post-menopausal breast	413,784	1,975,826	2,389,610	853,084	4,073,489	4,926,572
Uterus	1,131,745	5,404,100	6,535,845	2,277,280	10,874,048	13,151,328
Ovary	55,084	263,027	318,111	111,269	531,319	642,579
Cardiovascular and cerebrovascular diseases						
Ischemic heart disease	23,359,119	39,929,872	63,288,991	48,949,863	83,674,464	132,624,328
Ischemic stroke	4,160,043	7,111,142	11,271,185	8,625,915	14,745,063	23,370,978
Hemorrhagic stroke	5,734,612	9,802,694	15,537,306	11,621,550	19,865,775	31,487,325
Diabetes	1,048,970,935	1,244,320,754	2,293,291,689	2,109,852,550	2,502,770,313	4,612,622,863
Chronic kidney disease	9,721,808	3,430,500	13,152,308	19,488,360	6,876,788	26,365,149
Low back pain	4,032,684	20,910,756	24,943,440	8,160,218	42,313,335	50,473,552
Age group						
≤14 years	43,508,259	52,224,382	95,732,641	70,748,253	85,017,154	155,765,407
15–34 years	96,446,377	117,832,929	214,279,306	196,793,417	240,110,909	436,904,326
35–54 years	440,466,289	539,275,782	979,742,071	748,261,431	920,537,258	1,668,798,689
55–64 years	257,354,285	318,606,611	575,960,896	651,993,607	801,635,275	1,453,628,883
65–74 years	169,776,207	209,156,540	378,932,747	362,301,762	447,133,353	809,435,115
75+ years	95,198,861	120,741,470	215,940,331	190,574,942	242,542,423	433,117,365
Sex						
Women	397,680,399	493,792,265	891,472,664	919,996,608	1,135,874,649	2,220,673,411
Men	705,069,878	864,045,449	1,569,115,327	1,300,676,803	1,601,101,724	2,736,976,373
Total	1,102,750,277	1,357,837,714	2,460,587,992	2,220,673,411	2,736,976,373	4,957,649,784

*TEI*, total energy intake

## Discussion

In this study, we revealed that free sugar consumption in Canada contributes enormously to chronic diseases, to costs for the treatment and management of these CDs, and to costs associated with loss of human capital. The estimated reductions in the disease burden if Canadians were to comply with free sugar consumption recommendations are substantial for all CDs but are particularly pronounced for diabetes. Adhering to the recommendation to limit free sugar consumption to less than 10% of TEI would result in a reduction of approximately 27.0% in the prevalence of diabetes. For the stricter recommendation (<5 % TEI), this reduction would reach as much as 44.8%. The economic burden attributable to free sugar consumption is also substantial for all CDs, with diabetes accounting for the bulk of these costs. For all CDs combined, adhering to the recommendation to limit free sugar consumption to below 10% of TEI and 5% of TEI would have avoided $2.5 billion and $5.0 billion, respectively, in direct and indirect costs in 2019.

This is the first study to reveal the economic burden of free sugar consumption in Canada. In previous work, using the same methodology and the 2015 CCHS-Nutrition data, we estimated the economic burden for not meeting established recommendations for whole grains to be $3.8 billion, for nuts and seeds to be $3.8 billion, for fruits to be $2.5 billion, for vegetables to be $1.7 billion, for processed meat to be $2.2 billion, for milk to be $666 million, for red meat to be $231 million, and for SSBs to be $830 million in 2018 (Loewen et al., 2019). Notably, our estimate of $2.5 billion for the free sugar recommendation that consumption should be below 10% of TEI is of similar magnitude to those foods with a high economic burden (fruits and processed meats). Our estimate of $5.0 billion for the stricter free sugar recommendation exceeds all above-mentioned estimates. In other words, more CDs will be prevented and more costs for treatment and management of CDs will be avoided if Canadians are to comply with this recommendation (free sugar below 5% of TEI) than with any other established dietary recommendation. In Korea, the costs from disease treatment and premature mortality caused by excessive SSBs consumption were estimated to be KRW$ 633 billion in 2015 (approximately $CAN 19.42 per capita per year) (Shim et al., 2019). These costs are much lower than our estimates of the economic burden: approximately $65.44 per capita per year for not consuming below 10% of TEI and $131.87 per capita per year for not consuming below 5% of TEI. However, comparisons with studies from other countries are complicated because of differences in dietary patterns, health care systems, free sugar definitions, and research methodology (Meier et al., 2017; Shim et al., 2019).

Our estimate of the economic burden for not adhering to the recommendation for free sugar consumption below 10% of TEI ($2.5 billion per year) is approximately three times higher than the estimate for not adhering to the recommendations for the SSBs intake ($830 million per year) (Loewen et al., 2019). For the stricter free sugar recommendation, the economic burden estimate ($5.0 billion per year) was about six times higher than that for SSBs. These comparisons suggest a proportionately larger impact of interventions targeting a broader set of products containing free sugar (e.g., confectionery, chocolate, and ice cream) as compared to interventions targeting SSBs and sugary drinks (Cobiac et al., 2017).

Using pricing strategies (food taxes and subsidies) is considered a key policy tool to reduce the chronic disease burden and associated health care costs (WHO Europe, 2015). Jones et al. projected that a 20% tax on SSBs would avoid $7.4 billion in health care costs in Canada between 2016 and 2041 (Jones et al., 2017). Coming on the heels of public health successes from taxation of tobacco cigarettes, taxation of SSBs is considered to be most effective in inducing health-promoting changes in sugar consumption and is recommended by the WHO and Dietitians of Canada to influence the demand for foods high in sugar (WHO Europe, 2015; Dietitians of Canada, 2016). Targeting SSBs has the practical advantages of focusing on a single product or an easy-to-define category of products that are energy-dense and nutrient-poor but with close healthier substitutes (e.g., water), and as such is administratively simple to implement. Where taxation of SSBs is a reality in over 40 countries and cities (Bridge et al., 2020), Canada and many other jurisdictions are currently considering this strategy to curb sugar consumption. However, having the sole focus on SSBs comes with the drawback that only a modest portion of all free sugar in the Canadian diet will be taxed: Liu et al. recently estimated that of all free sugar that Canadians consume, only 17.5% originates from SSBs (Liu et al., 2020). In other words, the targeted health gains arising from taxation will have to come from a movable margin of this 17.5%. Indeed, the WHO recommendation recognizes that SSB taxation should only be applied in settings where SSB consumption is a significant contributor to free sugars intake (i.e., greater than 20 L per person per year) (WHO, 2017).

Another drawback of targeting a single product or product group is that it allows consumers to choose alternative sources of free sugar that are not taxed and herewith circumventing the taxation objectives. The findings from this study provide support for broader taxation of a wider range of foods and beverages high in free sugar, which has the potential not only to reduce free sugar consumption at the population level but also to improve the overall quality of the diet (WHO Europe, 2015). Several countries have or had implemented policies with taxation targets beyond SSBs. For example, Finland, Norway, and Hungary had introduced taxation of sweets, chocolate, ice cream, and other sugar-containing foods in addition to SSBs (WHO Europe, 2015). Although administratively complex, a comprehensive set of pricing policies that includes a broad tax on free sugar content (e.g., a given amount per 100g of sugar contained in certain products), and an excise duty on specific products containing sugar (e.g., a given amount per kg/L of the specific product) seems to be needed to reach more consumers and to curb their sugar consumption.

Finally, building on lessons from successful tobacco control, a comprehensive package of complementary policies in addition to taxation is advocated to effectively reduce sugar consumption at the population level (Dietitians of Canada, 2016; WHO, 2017). Taxation of SSBs has been shown to be financially regressive whereby low-income groups bear a larger tax burden (Kao et al., 2020; Men et al., 2021), calling for policies that sugar tax revenues be reinvested in the production, distribution, and marketing of healthful foods to support food security for these low-income groups (Men et al., 2021). Other complementary policies may include regulatory measures (e.g., front-of-package labelling, regulation of health claims, and advertising), legislation limiting or banning use of free sugar across the food supply chain, industry incentives for product reformulation, supportive environments in public institutions (e.g., hospitals, schools, nursing homes) to serve low sugar meals, health education campaigns, and dietetic counselling of people at higher risk (Sassi, 2016; WHO, 2017). Yet, other interventions, including public awareness education initiatives and product labelling policies, have also had SSBs as their only target in Canada (CDA, 2020; Fung et al., 2013).

In the present study, we observed that men contribute much more to the economic burden of excessive free sugar consumption as compared with women, which is consistent with previous reports on the economic burden associated with unhealthy eating (Ekwaru et al., 2016; Krueger et al., 2011; Lieffers et al., 2018; Loewen et al., 2019). Though men consumed more free sugar in absolute terms (grams per day), free sugar consumption as a share of TEI was similar for women and men. The observed sex differences in the economic burden are thus not a result of sex differences in compliance with free sugar recommendations. Instead, they originate from a higher prevalence of chronic diseases, and specifically diagnosed and undiagnosed diabetes, among men relative to women (Leong et al., 2013), and the ensuing higher economic costs attributable to diabetes among men relative to women (American Diabetes Association, 2018). Complementary policies that promote healthy eating and active lifestyles may reduce the prevalence of diabetes and other chronic diseases, and herewith their economic burden and the impact of free sugar on this economic burden. Where these complementary policies specifically target men or are more effective among men than among women, they will reduce the current sex differences in economic burden.

The present study has several strengths. We used the established free sugar definition by the WHO, the 2015 CCHS-Nutrition, Canada’s most comprehensive dietary survey of the past decade, and robust estimates of free sugar consumption (Liu et al., 2020). With respect to the latter, we had considered the free sugar content of each of 5374 foods and beverages recorded in the 2015 CCHS-Nutrition (Liu et al., 2020). We had used both the first and second 24-h recall and had applied the recommended bivariate NCI method so that our estimates are representative for the Canadian population (Liu et al., 2020). We believe our estimates of free sugar consumption are therefore more robust than those obtained through an alternative approach based on the public use microdata file which does not include the second 24-h recall and does not allow the application of the bivariate NCI method, and considered the free sugar content of 177 foods and food groupings (Wang et al., 2020). In the absence of established risk estimates for CDs associated with consumption of free sugar in our diet, we assumed that free sugar in our diet exhibits the same risk as the equivalent amount of free sugar in SSBs. Future research, however, has to reveal the extent to which this assumption is correct. As a limitation to this study, we should mention that dietary intake is obtained through self-report, which is prone to error. Another limitation is that our economic burden estimates represent underestimations. For the direct health care costs, we considered only hospital, physician, and drug costs associated with 16 CDs and not, for example, costs associated with dental caries, mental health, and other diseases. Also, the economic burden following the COVID-19 pandemic will likely increase further since people with CDs (diabetes, hypertension, cardiovascular and cerebrovascular disease, chronic obstructive pulmonary disease, cancer) were 2–4 times more likely to have severe COVID-19 symptoms and complications, thus increasing the health costs for ICU admission and hospital stays (Roncon et al., 2020; Williamson et al., 2020). The public health measures implemented to contain the spread of the virus (i.e., lockdowns) have also increased unhealthy lifestyle behaviours, including free sugar intake (WHO, 2020).

## Conclusion

The magnitude of the public health and economic burden attributable to excessive free sugar consumption sounds an alarm and exposes an area of urgent need for action. Public health interventions to reduce sugar consumption must go beyond taxation of SSBs to target a broader set of food products. Public health interventions must also extend beyond taxation to comprise a comprehensive suite of complementary approaches in order to more effectively reduce the public health and economic burden of CDs.

## Contributions to knowledge

What does this study add to existing knowledge?

- This is the first study to estimate the economic burden of excessive sugar consumption from all foods and beverages in the Canadian diet. To date, such estimates only existed for sugar in SSBs.
- If Canadians were to comply with established recommendation (free sugar energy below 10% of total energy intake (TEI)), an estimated $2.5 billion per year in direct health care and indirect costs could have been avoided. For the stricter recommendation (below 5% of TEI), this was $5.0 billion per year.
- The economic burden of free sugar in the Canadian diet is 3 to 6 times that of SSBs.

What are the key implications for public health?

- Pricing strategies targeting SSBs have the practical advantages of focusing on a single product or an easy-to-define category of products that are energy-dense and nutrient-poor. These strategies will be limited in their effectiveness of reducing the public health and economic burden associated with free sugar consumption because only 17.5% of free sugar in the Canadian diet is from SSBs.
- Public health interventions to reduce sugar consumption should therefore go beyond taxation of SSBs to target a broader set of food products and to include complementary approaches, in order to more effectively reduce the public health and economic burden of CDs.
